# Mpox Virus as a Global Public Health Emergency: A Scoping Review

**DOI:** 10.1155/cjid/6683501

**Published:** 2025-01-22

**Authors:** Sathish Sankar, Pachamuthu Balakrishnan, Yean K. Yong, Sivadoss Raju, Vijayakumar Velu, Esaki M. Shankar, Marie Larsson

**Affiliations:** ^1^Department of Microbiology, Centre for Infectious Diseases, Saveetha Dental College and Hospitals, Saveetha Institute of Medical and Technical Sciences, Chennai 600077, Tamil Nadu, India; ^2^Department of Research, Meenakshi Academy of Higher Education and Research (MAHER), Chennai 600078, Tamil Nadu, India; ^3^Laboratory Center, Xiamen University Malaysia, Sepang 43900, Selangor, Malaysia; ^4^Kelip‐Kelip! Center of Excellence for Light Enabling Technologies, Xiamen University Malaysia, Sepang, Selangor, Malaysia; ^5^State Public Health Laboratory, Directorate of Public Health and Preventive Medicine, DMS Campus, Teynampet, Chennai 600006, Tamil Nadu, India; ^6^Department of Pathology and Laboratory Medicine, Division of Microbiology and Immunology, Emory National Primate Research Center, Emory Vaccine Center, Emory University School of Medicine, Atlanta 30329, Georgia, USA; ^7^Department of Biotechnology, Infection and Inflammation, Central University of Tamil Nadu, Thiruvarur 610005, Tamil Nadu, India; ^8^Department of Biomedical and Clinical Sciences, Division of Molecular Medicine and Virology, Linköping University, Linköping 58183, Sweden

**Keywords:** global health, monkeypox, Mpox, outbreak, public health

## Abstract

The monkeypox (Mpox) virus has emerged as a global public health emergency of international concern recently. The virus that was endemic in West and Central Africa has now been reported with chains of global transmission to several countries. A scoping review was carried out from the relevant literature available from PubMed, Scopus and Web of Science. This comprehensive analysis describes the virus epidemiology, pathogenesis, clinical manifestations, complications including secondary bacterial infections, diagnosis, treatment and vaccination. The article underscores the significance of key viral and immune mediators of infection and discusses updated recommendations on therapeutic strategies and vaccination.

## 1. Introduction

Monkeypox (Mpox) virus is a double-stranded DNA virus belonging to the genus Orthopoxvirus of the family *Poxviridae* and causes zoonotic infections. Mpox was declared a global public health emergency of international concern (PHEIC) by the World Health Organization (WHO), Geneva, in August 2024. The virus appears to have been primarily confined to the tropical rainforest regions of Central and Western Africa. Having remained largely unrecognized until 1760, the first description of the infection originated from monkeys of the Brazilian forest showing lesions akin to smallpox pustules followed by several reports during 1850–70. The virus was placed within the group of poxviruses in 1959 [1].

The first confirmed global outbreak of Mpox was reported in Denmark in 1958 among *Cynomolgus* monkeys imported from Singapore followed by outbreaks documented in the Netherlands, the United States, the Philippines, Malaysia and certain other countries [2]. Nevertheless, as the infections were largely confined among primates, the absence of human infections during these outbreaks misled the transmissibility of the virus, and humans were considered nonsusceptible [1, 3]. The Mpox virus was antigenically related to other poxviruses including smallpox, cowpox, camelpox, buffalopox and rabbitpox viruses as reported in the 1960s, based on morphology, nature of inclusion bodies (Guarnieri Type B), clinical manifestations and serological reactions [4]. The pathogenesis largely resembled smallpox and cowpox except the cutaneous haemorrhagic lesions that are produced in rabbits. Subsequently, human Mpox virus infection was reported in six individuals between late 1970 and early 1971 across three different West African regions [5]. At least four of these were confirmed with culture, hemagglutination and neutralizing titres with the poxvirus group. This confirmed the susceptibility of humans to Mpox and that vaccination against smallpox gave protection against Mpox [6]. During the same time, at least five species of rodents were found to be susceptible to Mpox virus, of which rabbits and white mice were highly susceptible, as the Mpox-specific antibodies were detectable in these species for over a year [7].

**Figure 1:**
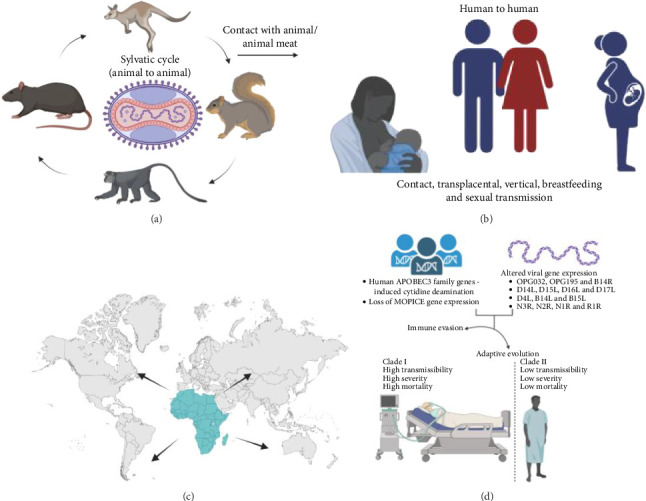
Mpox transmission. (a) The sylvatic cycle of Mpox transmission among the natural reservoirs. The virus is acquired by humans by contact with animals and/or animal meat; (b) the virus is transmitted among humans through skin and mucosal contact, breastfeeding and from mother to foetus; (c) the infection is endemic in Central and West Africa is now spread across 117 countries; (d) major host cell and viral factors play a crucial role in immunopathogenesis towards immune evasion and adaptive evolution.

When the smallpox eradication programme ended in Africa, sporadic outbreaks of Mpox infection were documented from the tropical rainforest regions of Central and West Africa (Figure 1). The animal reservoir of these infections remains essentially undiscovered, and most of the patients with documented Mpox had been in close contact with unvaccinated individuals [8]. A specific large outbreak in different regions of Zaire (now the Democratic Republic of Congo) during 1980–1985 among all age groups led to the revival of surveillance programs on zoonotic diseases [9] and presumed human-to-human transmission [10]. This outbreak also confirmed that Mpox infection occurred largely among unvaccinated contacts. The virus emergence and re-emergence in Zaire served as a model to study the disease pattern, incidence and attack rates, disease surveillance and establishment of laboratory diagnosis modalities such as ELISA for the detection of neutralizing antibodies [11].

The genomic compositions for smallpox, cowpox and Mpox were first reported in 1990 and revealed considerable similarity in genomic regions that determine the host range [12]. The evolution of PCR assays helped in differentiating further as distinct sequences among the poxviruses [13, 14]. In 2000, Mpox was declared a potential biowarfare agent, and the Mpox infection was considered an emerging zoonotic disease with public health importance, and several antiviral drugs were reviewed for likely repurposing and patient management [15, 16]. The infection was considered endemic in the rodent population in parts of West Africa. In 2003, imported rodents, exotic pets and Prairie dogs (*Cynomys* sp.) that were used in hunting caused an outbreak in different regions of the United States (Illinois, Indiana, Kansas, Missouri, Ohio and Wisconsin) [17–19]. The imported rodents included African rope squirrels (*Funisciurus* sp.), tree squirrels (*Heliosciurus* sp.), African giant pouched rats (*Cricetomys* sp.), brush-tailed porcupines (*Atherurus* sp.), dormice (*Graphiurus* sp.) and striped mice (*Hybomys* sp.). The smallpox vaccine helped prevent the spread of orthopox viruses and even outweighed the neurological complications the vaccine can give rise to as a side effect. Considering the surge in human-to-human transmission rates, especially among close contacts and healthcare workers, following the global lack of smallpox vaccination, the Mpox virus was placed on the red alert list, leading to a revival of antiviral therapies and vaccination programs [20, 21].

The first immunological evidence of protection against the Mpox virus by the smallpox vaccine focussed only on cross-reactive neutralizing antibodies but not cell-mediated immune responses [22]. Later, the CD8+ T-cells were reported to complement the antibody-mediated viral killing necessary for developing resistance against the infection [23]. The Mpox virus was distinguished as two distinct clades: Clades I and II. The virus types that are identified in Central Africa, especially in the Democratic Republic of Congo, were categorized as Clade I, which primarily afflicted children and adolescents [24]. The virus types that caused an outbreak in 2003 in the United States were categorized as Clade IIa and those that caused the 2017–2019 Mpox outbreak in Nigeria and the 2022 global outbreak were labelled as Clade IIb. During this global outbreak, the Clade IIb Mpox caused ∼99,000 confirmed cases and approximately 170 deaths in more than 117 countries (https://www.cdc.gov).

## 2. Epidemiology

### 2.1. Prevalence Data-Global Trends

The Mpox virus has become the most common and aggressive orthopoxvirus in the human population after the smallpox virus that was successfully eradicated in 1979. In the backdrop of waning immunity to the smallpox virus attributed to the global stop of vaccination, there is a universally acceptable consensus on the resurgence of related viruses.

In 2022, sporadic cases of Mpox from different countries outside of Africa were reported including the United Kingdom, the United States, Singapore and Israel. Most of them had a travel history to endemic areas or contact with individuals diagnosed with Mpox. The Mpox virus strains were identified as variant Clade I, which is considered to have a low fatality rate (1%). Soon, countries including Portugal followed by Canada, Spain and other European countries such as Belgium, Sweden, Germany, France, Canary Islands, the Netherlands and Italy reported a surge in Mpox cases. The WHO declared by mid-2023 a ‘PHEIC' considering the large outbreaks in several countries. By the end of 2023, the outbreak amplified exponentially to as high as 58,000 laboratory-confirmed cases and 20 deaths that spread to Asian countries including Hong Kong, China, the Philippines and Taiwan.

Currently, an alarming escalation in the global incidence of Mpox infections is being reported. Between 1 January 2022 and 31 July 2024, the total reported Mpox cases from 121 member states across all six WHO regions are 103,048 laboratory-confirmed cases and 229 deaths as per the WHO recent report (https://worldhealthorg.shinyapps.io/mpx_global/accessed on 12 September 2024). It is interesting to note that the majority of Mpox-infected (> 95%) and Mpox-related deaths (> 94%) documented were from regions that had no historical reports. The Mpox impact in the West and Central Africa corresponded to historical reports. This indicates that the reported sporadic cases during this outbreak are mostly linked to travel history and animals imported from the endemic regions. The most affected countries globally since January 2022 include the United States (*n* = 33,556), Brazil (*n* = 11,841), Spain (*n* = 8104), Democratic Republic of Congo (*n* = 4395), France (*n* = 4283), Colombia (*n* = 4256), Mexico (*n* = 4132), the United Kingdom (*n* = 4018), Peru (*n* = 3939) and Germany (*n* = 3886). All these countries account for 80% of the cases reported, globally as per the WHO. Among the confirmed cases, African Americans were highly affected (32.2%) followed by Hispanics or Latinos (31.03%), Whites (29.75%) and Asians (2.87%). The global trend also indicated that men aged 31 to 35 years are affected to a higher extent compared to other gender and age groups. The endemic Mpox infection peaks generally during the months of May and June and is the lowest from August to October (https://www.cdc.gov). In the USA, as of mid-January 2024, 32,063 cases and 58 deaths have been reported due to Mpox Clade II subtype. California reported the highest number of cases (6 160) followed by New York (*n* = 4528), Texas (*n* = 3163), Florida (*n* = 2992) and Georgia (*n* = 2075). A vast majority of the populations in these locations were either unvaccinated or had received only one dose of the smallpox vaccine. In the United States, the 2022 outbreak of Clade I Mpox viruses ended with ∼30,000 confirmed cases. The 2024 outbreak was caused by the Clade II virus, which is responsible for the ongoing worldwide outbreak since July 2024 (https://www.who.int).

**Figure 2:**
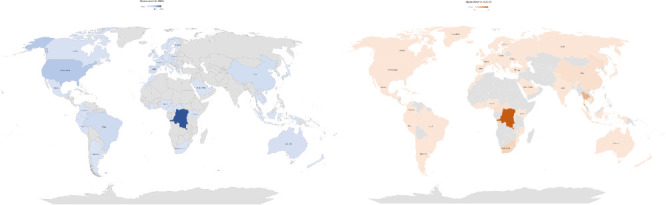
Mpox cases and deaths during 2024. As of September 10, 2024, a total of 6988 cases and 43 deaths were reported across 107 countries.

The first Mpox case in the WHO Southeast Asian region was reported from India in July 2022, and this was an individual who had a travel history to the Middle East. In a period of two to three months, at least 20 Mpox cases were reported thereafter from across different parts of Southeast Asia, predominantly from India followed by Thailand and Indonesia [25]. In July 2024, three major states of the Democratic Republic of Congo—Burundi, Rwanda and Uganda—reported confirmed cases with Clade I Mpox viruses. Although the viruses are endemic in these regions, the outbreak was more widespread across neighbouring countries and has led to stringent public health measures for prevention and response. Clade I is reported to be associated with a higher rate of severe disease as compared to Clade II [26]. The outbreaks that were reported from different countries from 1958 to 2023 are listed in Table 1, and the recent outbreaks in 2024 are shown in Figure 2.

### 2.2. Zoonotic Transmission

Primarily being a zoonotic disease, the natural animal reservoirs of the Mpox virus largely remain enigmatic. The initial detection of Mpox in diseased monkeys was followed by rope and sun squirrels (*Funisciurus anerythrus*) and other wild rodents. The infected animal likely shows symptoms such as fever, rashes, lethargy, loss of appetite, coughing, bloating, nasal/eye secretions and skin blisters (https://www.cdc.gov). Although many mammals could be susceptible to Mpox, there are very limited data available on the complete viral host range. The susceptibility among the species or varieties or strains that can affect each animal species largely remains ambiguous [27]. A retrospective observational study on animal reservoirs indicated four animal species including the African striped squirrel (*Funisciurus* sp.), African dormouse (*G. crassicaudatus*, *G. lorraineus*) and Gambian pouched rat (*C. gambianus*) acting as major animal reservoirs for Mpox [9].

Humans acquire Mpox infection from animals through direct contact with the body fluids, ingestion of infected or contaminated meat, animal bites and scratches. The virus is transmitted from infected humans through direct skin or lesion contact, respiratory droplets or large water droplets during prolonged face-to-face and sexual contact [28]. Furthermore, transmission can also occur via fomites contaminated with infectious material derived from infected lesions. Vertical transmission from the infected mother is not clear, but adverse foetal outcome was reported in an infected mother [29]. A recent meta-analysis indicated a high prevalence of Mpox DNA in seminal fluids (72.4%), indicating the potential role of sexual transmission. The rate of PCR positivity was higher in skin samples (89%) followed by anogenital/rectal samples, but lower in nasopharyngeal, urine and blood/plasma samples [30]. During the 2022 outbreak, 91% of the infected individuals tested positive for Mpox virus in their seminal fluids. However, horizontal transmission through genital secretions during sexual activity remains unclear despite the evidence of infections acquired following sexual contact. There is also considerable evidence that Mpox infection inflicts a considerable degree of tissue damage in the rectal compartment, leading to severe proctitis [31–33].

The use of experimental animal models represents a key strategy for studying the transmission dynamics and pathophysiology of Mpox virus infection in animals. Despite the serological and molecular evidence of virus infection in certain animals is it important to use correct models [26]. The prairie dog model resulted in similar pathological changes characterized by skin lesions and lymphoid tissue necrosis as seen in humans. Viral shedding was higher in the nasal secretions of the infected animals followed by spleen and cutaneous lesions. The lymphatic spread followed by hematogenous spread occurred resulting in secondary viremia [34]. The squirrel model showed similar results in the viral shedding being the highest in the oral secretions but did not reveal the involvement of the liver or spleen [35]. The Gambian pouched rat model indicated the highest viral shedding in oral secretions and indicated clinical signs of the disease [36].

### 2.3. Travel-Associated Mpox Outbreaks

The Democratic Republic of Congo recorded a high outbreak in 2023 with Clade I Mpox followed by the other outbreak in April 2024 with the same clade. International travel and social gatherings play an important role in Mpox transmission and so the neighbouring countries of the Democratic Republic of Congo are at a high risk of Mpox infection outbreaks. Hitherto, the countries bordering the eastern parts of the Democratic Republic of Congo including Burundi, Rwanda and Uganda have reported several cases of outbreaks due to Clade I Mpox suggesting transmission to neighbouring countries [37].

Mpox vaccination has been recommended for people at risk, which includes the unvaccinated population, pregnant women, neonates, individuals with other skin infections and immunocompromised individuals. Owing to the travel restrictions including commercial vehicles from and to the Democratic Republic of Congo, the risk of spread is limited (https://www.cdc.gov). Nevertheless, infection prevention and control as per the Centre for Disease Control and Prevention (CDC) guidelines are provided to reduce the risk of transmission and management of travel-associated Mpox. Travel to an afflicted country along with the onset of illness within 21 days of travel is considered an important epidemiologic criterion for probable disease confirmation (https://www.cdc.gov).

Vaccination recommendations, enhanced surveillance and public health measures play a key role in the mitigation of emerging Mpox. Travel to the countries that reported Mpox cases requires vaccination with a commercial JYNNEOS or ACAM2000 vaccine completed, with two doses at least 28 days apart. The second dose can be administered sooner if the individual is at risk [38]. However, the spread of Mpox cases with no evident travel links to any endemic areas, contact tracing and measures of infection severity are key epidemiological metrics, and even if challenging, it is essential to establish the public health policy [39].

### 2.4. Genomic Variants

The viral genome is a double-stranded DNA of about 196,858 base pairs in length coding for > 200 genes. The central part of the genome of about 101 kb is flanked by variable regions and inverted terminal repeats. The central region possesses conserved sequences coding for essential functions including transcription, replication, structural proteins and enzymes [34]. The virus shows antigenic similarities with other orthopox viruses, which provide cross-protection. Microevolution is believed to have emerged as a cause of hypermutations induced by apolipoprotein B mRNA–editing catalytic polypeptide-like 3 (APOBEC3), the enzyme that mediates cytidine deamination to uracil and also plays a vital role in antiviral immunity. The mutations in the specific viral DNA motifs could have implications for the evolutionary potential of Mpox during the outbreak [40, 41].

The difference in the virulence and transmission between the two Mpox clades are studied as it is vital for developing appropriate therapeutic strategies. The Clade I Mpox closely aligned with smallpox virus infections. Clade I was reported as the causative agent of a more severe form of the disease, mostly affecting the smallpox-unvaccinated group. Clades I and IIa were mostly endemic, but the Clade IIb infections occurred across the countries including the nonendemic regions with varied clinical presentation [42]. Clade II is connected to the transmission among sexual contacts especially among high-risk individuals such as men who have sex with men and people living with HIV. Clade II Mpox viruses also have a high human-to-human transmission without any apparent zoonotic link. Clade I Mpox cases were primarily among children and young men and had a high zoonotic transmission link [43].

The genetic characteristics of Clade IIb that separate it from Clade I and Clade IIa should be considered for further investigation on unique Mpox transmission potential. Mpox Clade I showed increased virulence with 2-logs higher lethal dose (LD50) than Clade II in animal models [44]. Significant differences in virulence were also observed among the Mpox clades with different routes of infection [24, 45]. The gene variations between Clades I and II that contribute to the severity of the transmission potential of the virus are not clearly understood, but it is believed that variable genes such as OPG195, B14R and OPG032 determine functions including host range, virulence and immunomodulation. Furthermore, the identical but inverted repeats of 6.4 kb sequences flanking at each end of the Mpox genome play a major role in immune evasion and host range. Insertion and expansion of inverted DNA repeats in the Mpox virus were reported to be a major factor in an outbreak in Germany [46].

Case-fatality ratio (CFR) associated with Clade I is about 10%, while Clade II has a CFR of around 1%. Compared to Clade I, the genes D14L, D15L, D16L and D17L are lost and the genes D4L, B14L and B15L are curtailed in Clade II. In addition, genes N3R, N2R, N1R and R1R that are present in Clade IIa are absent in Clades I and IIb. The role of these genetic changes in the pathogenesis mechanism is so far unknown [47].

## 3. Immunopathogenesis

### 3.1. Pathophysiology

The virus enters the host through various routes such as the mucosa overlying the oropharynx, nasopharynx, ocular, rectal and genitourinary tracts, and the skin. Although the virus primarily affects the lymphoid tissue, it has a broad cell tropism and therefore can afflict multiple organs [48]. The specific host cell receptors that mediate virus entry are not clearly defined, but virulence factors such as the Mpox inhibitor of complement enzymes (MOPICEs), a 24-kDa secretory protein and its homologue, complement control protein are reported to modulate the host range and tissue tropism [47, 49]. MOPICE is expressed by the highly virulent strains of Clade I, while Clade II lacks this protein. This protein is also found to modulate adaptive immune responses in the host [50].

Similar proteins that appear in variola and vaccinia viruses are called smallpox inhibitors of complement enzymes (SPICEs) and vaccinia complement control protein (VCP), respectively. These proteins regulate the human complement system, and their dimeric forms act as major virulence factors. These proteins show significant differences in their efficiency among the poxviruses [45]. This could be a major factor determining the host range, infectivity and transmission potential of different poxviruses. These complement regulators and other virulence factors such as IL-1 binding protein, B10R, B14R, B19R, D10L and D14L could play a vital role in the pathogenesis and tissue tropism [51, 52]. Further studies on the differences in the structural and functional motifs of these proteins could help with our understanding of the broad tissue and host tropisms of Mpox viruses. The virus possibly uses multiple ligands to interact with the cell surface receptors, which may also explain the broad animal and human host cell tropism and the challenges in the identification of specific receptors used by the virus to infect. Multiple receptors including heparan sulphate, glycosaminoglycans and chondroitin have been reported to mediate virus entry [53]. The viral genome expresses 25 membrane proteins that are species-specific and contribute to the pathogenesis based on their presence or modifications in these proteins [54]. The membrane proteins vary in composition within the two distinct infectious poxvirus particles viz. extracellular enveloped virions (EEVs) and intracellular enveloped virions (IEVs) [34].

After gaining cellular entry, early, intermediate and late viral mRNA synthesis occurs followed by protein synthesis. The infectious virions are assembled and become an IEV as it is encased within a bilayer membrane generated from the Golgi complex including Golgi-associated retrograde protein and conserved oligomeric Golgi (COG) complex [50]. These also play a vital role in virus–host interaction and are part of the formation of EEVs for a successful egress process.

### 3.2. Immune Response and Host Interactions

The virus entry into a susceptible host cell is mediated by COG complex proteins. These proteins assist viral fusion and mediate the virus entry and early gene expression [53]. The viral-induced host immune response may play a significant role in the disease pathogenesis. The disseminated Mpox infection activates a strong immune response involving multiple organs including lymphoid organs [34]. The viruses also possess unique immune evasion strategies, leading to an increase in prevalence rates among vulnerable animal and human populations, and one of these strategies is the viral evasion of TLR3 signalling and through the downregulation of cytokines and chemokines such as TNF-α, IL-1*α* and CCL5 [55].

**Figure 3:**
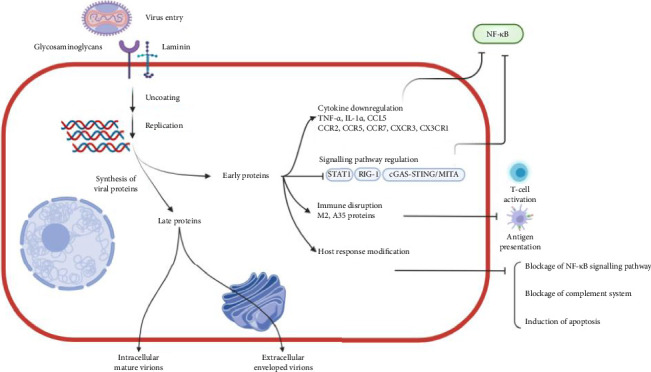
Life cycle and role of Mpox virus protein in the immunopathogenesis process. The pathogenesis process includes virus entry, fusion, replication and egress. The virus upon entry through various cell receptors such as glycosaminoglycans and laminins, undergo uncoating, replication and transcription in the cytoplasm. The early proteins include transcription factors required for replication, growth factors and immune defence molecules. The late proteins serve as structural proteins resulting in the production of intracellular mature virions and Golgi-wrapped extracellular enveloped virions during the replication cycle.

The innate immune responses act as the first line of defence and include cells such as monocytes and natural killer (NK) cells and innate factors such as Type I interferons and inflammatory cytokines. Adaptive immunity plays a substantial role in virus clearance and long-term protection. The efficacy of the NK cells is hampered by downregulating the chemokine receptors such as CCR2, CCR5, CCR7, CXCR3 and CX3CR1 required for NK cell migration [56, 57]. The most notable innate mechanism activated is the cGAS-STING/MITA pathway. The pathway mediates immune responses against many DNA viruses, bacteria, parasites, autoimmune diseases and cancers. This pathway has emerged as a crucial element of innate antiviral immunity. The viral F17 protein interferes with the cGAS-STING/MITA signalling pathway and sequesters the regulators of mammalian targets of rapamycin complexes mTORC1 and mTORC2 [58] and through other nuclease enzymes termed as ‘poxins' that cleave 2′3′-cGAMP to control cGAS-STING/MITA signalling [53]. Many other viral genes including vaccinia virus protein 018 and E3 protein inhibit IFN-signalling pathways, and F14 protein disrupts NF-κB activation [59]. Regarding viral effects on the activation of the adaptive response, the M2 protein and A35 protein disrupt antigen presentation, T-cell priming, and chemokine synthesis and function together in modulating the host immune responses (Figure 3) [50].

## 4. Clinical Manifestations

### 4.1. Pathological Features

Following an incubation period of 9.5 days, the virus replicates in the local inoculation site and spreads to local lymph nodes. The symptom onset resembles smallpox infection with early symptoms of painful lesions with fever and other prodromal syndromes that last for 1–4 days including chills and lymphadenopathy. Moreover, the symptom of myalgia occurs either before or after the appearance of lesions [28]. Lymphadenopathy is considered an important clinical manifestation and the pathognomonic sign of Mpox that could help distinguish it from other orthopoxvirus infections and other skin diseases [60]. Rashes appear within 3 days of fever, and the common skin sites include the face, chest, arms, legs, genitals, anorectals, palms, soles, mouth and eyes. The rash is predominantly monomorphic in a centrifugal distribution and develops into vesicular and pustular lesions that last for 5–7 days; thereafter, they become crusts and peel off within a 15–22-day period [61]. Based on the skin lesions, the severity score can be classified as mild (< 25 lesions), moderate (25–99 lesions), severe (100–250 lesions) and very severe (> 250 lesions) (https://www.who.int). Hospitalization is not required in most cases, the treatment largely includes attentive monitoring, antiviral therapy and management of secondary infections and associated complications (https://www.cdc.gov).

### 4.2. Other Manifestations

Mpox is foremost a self-limiting disease but can give extended clinical manifestations involving multisystems, which are associated with fatal outcomes, especially among the high-risk groups [62]. Immunocompromised individuals, individuals with pre-existing skin eczema, and children and HIV infection are considered risk groups for developing life-threatening Mpox. Mpox can, apart from giving vesiculopustular lesions on the skin and oral mucosa, cause oral, ocular and neurological manifestations, acute renal injury, fulminant myocarditis leading to pericardial effusion, and pericarditis and lymphadenitis in the cervical, submandibular and inguinal regions [63–66].

Oral manifestations primarily are based on sexual behaviour and mainly present sore throat, ulcers, vesicles and dysphagia. Signs and symptoms in the pharynx or oropharynx, especially the tonsils, tongue and perioral site, are predominantly reported followed by lips and palate. The key clinical features resemble other viral and bacterial infections such as Herpes viruses, Coxsackie viruses and *Streptococcus pneumoniae*, presenting oral lesions with sore throat and should be considered for differential diagnosis [67]. It is noteworthy that the appearance of oral lesions following sexual contact should be considered highly suspicious as they were increasingly observed among individuals who had oral sex with Mpox-infected individuals [67].

Anogenital lesions and skin rashes with lymphadenopathy were predominantly seen among the infected during the 2022 outbreak. Other sexually transmitted infections such as syphilis and gonorrhoea should be considered for differential diagnosis as these pathogens cause pharyngeal infections as well as maculopapular lesions in the genitals along with fever and lymphadenopathy [68].

The neurological symptoms range from mild headache and myalgia to encephalitis. More severe cases presented decreased general responsiveness, rigidity and bilateral Babinski signs, which are common neurological presentations associated with many neurodegenerative diseases and pathogens infecting the central nervous system [69]. Apart from these, Mpox-associated ocular infections can lead to keratitis and conjunctivitis, and this can lead to decreased vision [69].

### 4.3. Secondary Bacterial Infections

Several bacterial pathogens mimic Mpox infection due to their clinical manifestations and could result in underreporting or misdiagnosis, which could further increase morbidity and mortality or predispose to outbreaks. Secondary bacterial infections occur with Mpox with a prevalence of 30%–75% and are further complicated by increasing the severity of Mpox infection [70]. Notably, pharyngeal coinfections with Group A streptococci have been frequently reported during the 2022 outbreak. The infected patients exhibited posterior oropharyngeal erythema, uvula oedema and vesicular lesion on the epiglottis. These patients respond to antibiotics but require lengthy hospitalization with serious complications. It is therefore important for clinicians to monitor for oropharyngeal manifestations especially when corticosteroids are administered [71, 72]. Moreover, *Streptococcus* infection in HIV-infected has been reported with multimucosal involvement and systemic infection, indicating its importance among patients with comorbidities. Patients with secondary bacterial sepsis during Mpox infection succumb to polymicrobial infection, septic shock with multiorgan involvement, and death [72].

The secondary bacterial infections could be mild and localized but can become complicated if early identification and clinical management are not done with appropriate antibiotics. Mpox-associated genital lesions are commonly seen in patients infected with sexually transmitted infections. These infections lead to further complications such as rectitis and proctitis in men and infertility in women. MSM usually presents lesions in the anogenital areas and frequently reports coinfections with *Neisseria gonorrhoeae* and *Chlamydia trachomatis* [73] and *Treponema pallidum* [74]. Coinfection with such sexually transmitted pathogens results in delayed clearance of Mpox, leading to prolonged viral shedding and decelerating therapeutic drug response [70].

### 4.4. At-Risk Populations

People living in the endemic regions of Africa, or neighbouring areas, and individuals having close contact with the affected communities have a high risk of acquiring the disease. Close physical contact including sexual and nonsexual interactions is of serious concern, especially in the adult population [75].

The lack of or incomplete smallpox vaccination is a major factor associated with Mpox [76, 77]. Prior immunization for smallpox is reported to protect from Mpox with up to 80.7% efficacy and decreases the risk 5.2-fold [78]. According to the European CDC, the overall risk of the disease is low; however, among high-risk groups such as those underlying immunocompromised conditions due to HIV infection, pregnant women and newborns are reported with moderate or high risk (https://www.ecdc.europa.eu/). The other risk factors include handling, hunting and consuming rodents, contact with wildlife, shared drinking, sleeping and eating and coinfection with large skin lesions, patients lacking sufficient data and patients with chronic skin conditions such as atopic dermatitis and acute skin conditions such as burns [79].

## 5. Transmission Dynamics

### 5.1. Simulation Model and Parameter Estimation

Estimation of transmission potential from the high-risk group, men having sex with men (MSM) to non-MSM was analysed using a multigroup dynamic model. The possibility of contact Mpox infection from an individual with a high-risk sexual behaviour was less than 10^−5^, the cumulative incidence rate was about 6 × 10^−8^, the required vaccine coverage was 80%, and the simulated R_0_ was 2.13 [80].

A recent study analysed transmission dynamics through mathematical modelling among different high-risk sexual behaviour groups including MSM, gay and bisexual individuals. The study indicated an incubation period of 6.9 days and R_0_ ranging from 1.41 to 2.17. The study also showed MSM to be the group with the highest risk and emphasized the efficacy of vaccination in reducing the rate of infection [81]. Sexual transmission is reported to be the major route of spread compared to nonsexual contact transmission such as through respiratory particles and contaminated surfaces. During the 2022 outbreak, among different modes of transmission, male-to-male sexual contact had been the most significant mode that appears to have increased the risk of disease acquisition. This could be possibly due to certain sociobehavioural correlates in the population. Another study identified the superspreader gatherings and sexual networks particularly among MSMs as a major risk factors for the spread of Mpox. The time-discrete susceptible-exposed-infectious-recovered (SEIR) model estimated *R*_0_ of 2.25 to 2.7 when different factors including travel history, exposure settings and transmission routes were analysed [82]. A systematic review of mathematical models on Mpox transmission indicates several parameters including human-to-human contact/transmission rate, progression rate, recovery rate and death rate. The study reported an *R*_0_ of 0.3 and an effective reproduction number *R*_*e*_ of 0.08 [83].

Mpox cases are considerably higher among MSMs due to their specific sexual patterns. This includes having sexual contact with multiple partners, engaging in interconnected sexual networks and sexual events such as Chemsex parties and social stigma associated with MSM significantly [75]. These markedly increase the risk of Mpox disease transmission. Massive super spreading events are linked to crowding within confined spaces, repeated or new sexual contact and coinfections such as HIV. Another study showed the potential increase in Mpox spread in the population associated with HIV endemicity. Mpox spread has been reported to increase in the presence of HIV and is therefore applicable to MSM population [84]. Among MSMs, the presence of primary lesions in the external genitalia and anus and subsequent contact with the lesions during the sexual practices further facilitate the disease transmission within the MSM population [85].

A Bayesian susceptible-infected-recovered (SIR) model was used to estimate the transmission dynamics, which showed an *R*_0_ of 0.817 to 2.251 with an infectious period of 8.5 days. The ratio of *R*_0_ and effective reproductive number (*R*_*e*_) ranged from 1 to 1.46 and indicated the required vaccine coverage of 37% [86]. The *R*_*e*_ value indicates the average number of secondary cases per infectious case in both susceptible and protected populations, while *R*_0_ indicates the number of persons infected by infectious persons in the susceptible population. The values of both *R*_0_ and *R*_*e*_ help in predicting a pandemic outbreak using contact-tracing data and mathematical models [87].

Hence, these different mathematical models indicate varied *R*_0_ and *R*_*e*_ values that make prediction more unreliable and clearly demonstrate that it is important to include several epidemiological factors to calculate an accurate prediction of transmission dynamics.

## 6. Outbreak Management

Mpox represents a significant public health concern and warrants a robust public health initiative on emergency response management targeting enhanced preparedness, rapid diagnosis, strengthened epidemiological surveys, effective interventions and vaccination. The Korean Disease Control and Prevention Agency had recommended pre-exposure prophylaxis for high-risk groups [88].

The standard care during the Mpox outbreak situation could begin with individual testing and assessment to determine the risk group/s and the requirement of hospitalization, if not home isolation will be adequate. These cases shall undergo testing by PCR. Patients presenting severe illness or posing a risk to others should be immediately notified to public health for appropriate infection control. The PCR-negative patients shall be advised to undergo rest and isolation if the symptoms are evident and look for other differential diagnoses [89].

The WHO guidelines on the management of Mpox patients among high-risk groups recommend management with optimized supportive care interventions (https://www.who.int). Recommendations on the prevention and control measures are provided by the CDC for patients in a healthcare setting. This includes additional precautions to prevent transmission with appropriate infection control practices in place, patients placed in individual rooms, use of personal protective equipment (PPE) and waste management (https://www.cdc.gov).

Environmental infection control in healthcare facilities including soiled linens, clothing and bedding are handled following the specific guidelines of the CDC (Guidelines for Environmental Infection Control in Health-care Facilities) with special care as they are implicated in disease transmission (https://www.cdc.gov). Among the healthcare workers, infection prevention measures should be implemented including hand hygiene, training on standard procedures on the use of PPE and provision of special care for pregnant and immunosuppressed individuals. Alongside standard infection control practices, patient isolation or self-isolation, use of PPE, personal hygiene, waste management and other environmental infection control protocols are advocated. The use of EPA-registered hospital-grade disinfectant with potential antiviral property, appropriate handling of soiled linens/surfaces and avoidance of activities such as sharing clothing, dry-dusting and sweeping have been recommended in the guidelines. Postexposure assessment of risk including 21-day monitoring period shall be followed in accordance with the recommended guidelines.

The current outbreak indicates a deficiency in sufficient and quick response to infections with the potential to cause outbreaks. The clinical outcomes for individuals in high-risk groups are of serious concern. Asian and Oceania countries with extremely concentrated and connected sexual contact networks are more vulnerable to transmission and require urgent management strategies to prevent outbreaks among them. Necessary screening and evaluation for sexually transmitted infections including HIV testing along with health education and improved accessibility should be a priority for substantial improvement in prevention and treatment.

## 7. Diagnosis and Treatment

### 7.1. Diagnostic Assays

Immediate testing for Mpox should be conducted on any suspected case. The result should be interpreted after careful consideration of associated clinical and epidemiological factors for assessing the likelihood of infection. Due to the atypical presentation of skin rashes, a diagnosis could be challenging, and hence, it is important to consider potential causes of skin rashes and differential diagnosis.

The appropriate clinical specimen for diagnostic confirmation includes skin lesion material such as swabs of lesion exudate, roofs of multiple lesions or lesion crust from the suspected cases. The collected swabs should be placed in a viral transport medium, and preferably, samples from lesions, crusts and vesicular fluids should be collected in separate tubes. Depending on the clinical presentation and availability of sufficient viral genomic material from the lesion, additional specimens including urine, semen, genital and rectal swabs and blood samples could be considered especially during acute viremia. It is recommended to collect the sample preferably during the prodromal phase and lesions during the macular stage by trained personnel. In the case of neurological involvement, PCR on CSF specimens should be considered [69].

PCR is the recommended confirmatory test for either generic identification of orthopox viruses or specific Mpox viruses. Viral culture is not recommended for a routine diagnostic procedure. Additional sequencing techniques might provide additional information on virus clade useful for epidemiological purposes [90].

### 7.2. Antiviral Drugs

There is no approved treatment for Mpox infections available, but tecovirimat or Tpoxx, a potent antiviral drug approved by the FDA for the treatment of smallpox was used successfully during the 2022 outbreak [91]. The efficacy of tecovirimat has been shown specifically beneficial, especially among high-risk groups, but this drug is not recommended for a mild infection. A reduction in the duration of Mpox lesions in children and adults was not achieved by the drug for Mpox infections caused by Clade I viruses; nevertheless, it significantly reduced the mortality rate from 3.6% to 1.7% [92]. The drug did not show any adverse effects, was tolerated well, and improved the clinical outcome at different scales of clinical severity [93]. To note, tecovirimat is still at the experimental stage being tested for its safety and efficacy for Mpox. The study of tecovirimat for Mpox (STOMP) is ongoing, and the result from this clinical trial is expected to be available soon. Additional strategies such as a combination of tecovirimat with other antiviral or antibiotics should be considered to treat specific or clinical manifestations such as systemic infections, and ocular and oral infections.

Brincidofovir, a prodrug of cidofovir is being used to treat severe cases of smallpox disease and has shown to be effective against Mpox viruses as well. The drug is recommended for adults and neonates who do not respond well to tecovirimat, are severely immunodeficient, or report severe disease or a high risk of progression to severe disease (https://www.cdc.gov).

Vaccinia immune globulin intravenous (VIGIV) is a licenced treatment for vaccinia-vaccine-induced complications. This therapy can be considered as prophylaxis for severely immunocompromised individuals or can be used in addition to tecovirimat or brincidofovir treatment of Mpox.

### 7.3. Vaccine Research

Advocacy for vaccination against the orthopox viruses dates back to 1798 when Edward Jenner demonstrated immunogenicity and cross-protection by cowpox virus (vaccinia) against smallpox virus. Dryvax was the first-generation smallpox vaccine, which was used globally and withdrawn in 2008. The ACAM2000, a second-generation live vaccine, is in use for certain at-risk populations. The JYNNEOS vaccine based on the modified vaccinia Ankara-Bavarian Nordic (MVA-BN) is a live attenuated, third-generation, smallpox vaccine developed by Bavarian Nordic, Denmark. A single dose of MVA-BN is recommended for adults and is shown to be highly effective among high-risk sexual contact populations. Apart from adults, the vaccine is highly effective in inducing humoral and cell-mediated immune responses in children as well [94]. A systematic review and meta-analysis on the efficacy and safety of MVA-BN indicate 75% efficacy with a single dose and up to 80% efficacy with a two-dose vaccine [95]. A single vaccination also showed high efficacy in protection as pre-exposure prophylaxis in HIV of a high-risk Spanish cohort population [96]. The JYNNEOS is administered subcutaneously in a two-dose series (0.5 mL per dose) 1 month apart or as a two-dose series as intradermal routes of administration (CDC, Atlanta, USA).

## 8. Conclusions

During the global health emergency, it is of utmost importance to focus on pandemic preparedness, efficient management of epidemics and strengthening international ties for combined efforts in diagnosis, treatment and prevention.

To implement the appropriate and effective countermeasures against emerging viruses, there is a need to enhance the laboratory testing facilities with wider testing platforms towards early detection. Considering the growing number of afflicted populations, joint international action for coordinated measures through evidence-based national and international policy guidelines. Several challenges remain in implementing public health policies, especially in resource-limited settings and regions with socioethnic issues. Reinforced surveillance measures, multisector partnerships, prophylactic measures, capacity strengthening, tackling social stigma, guaranteeing fair access to vaccines and healthcare services are a few strategies that can help overcome these obstacles. Isolation, active monitoring of high-risk individuals and effective vaccination strategies are the key public health measures to be implemented. Future research focussing on the long-term clinical impact of Mpox, outbreak preparedness, enhanced diagnostics and effective vaccines is imperative. The use of the two vaccines recommended by the WHO Advisory Group of Experts on immunization and national regulatory bodies shall be accelerated for all countries, especially for lower-income countries. A such harmonized battle along with public health professionals, similar to the handling of COVID-19 pandemic, could help mitigate Mpox infection successfully.

## Figures and Tables

**Table 1:** Outbreaks of Mpox viruses stratified by cases, deaths, source of infection and major clinical manifestations.

Study population	Year	Age (years)	Number of cases	Number of deaths	Vaccination	Source of infection	Clinical presentation
Monkeys	1958	—	10 outbreaks	—	—	Monkeys imported from different countries	—

Waka Bokeka	1968	—	70	18	—	—	—

Liberia, Nigeria and Sierra Leone	1970-1971	4, 6, 9 and 24	Five	0	Unvaccinated	Possible contact with household rodents and bats (one case), familial contact (four cases), not established (one case)	Afebrile, vesiculopustular rash

DRC	1970	9 months	1	0	—	Familial contact with a history of monkey carcass consumption	Haemorrhagic lesion, centrifugal distribution, otitis, mastoiditis
	1970–1975	—	20	3	2 Vaccinated	NA	

DRC	1982	6 months	1	0	Unvaccinated	Possible contact with chimpanzee bite	Pustular rashes, bilateral inguinal lymphadenopathy

DRC	1983	1–7	5	1	1 Vaccinated, 3 unvaccinated, 1 unknown	Possible contact with monkey meat (one case) and close contact with the index case (four cases)	Ever, skin rash, oral enanthem, centrifugal distribution, lymphadenopathy

DRC	1980–1984	< 15	214	—	16 Vaccinated and 46 unvaccinated	130 primary cases (contact with wild animals), 22 coprimary and 62 secondary cases	Skin lesions

DRC	1980–1985	0–40	282	27	32 Vaccinated and 250 unvaccinated	NA	—

DRC	1996	< 25	11	6	Unvaccinated	Familial contact	—

DRC	1996-1997	—	115	5	20 Vaccinated	NA	Cervical lymphadenopathy, sore throat, mouth ulcers, cough, diarrhoea, facial vesicular–pustular rash

DRC	1996-1997	> 25	338	33	—	Consumption of animal meat	Lymphadenopathy, crusty skin lesions, rash on the soles and palms, coinfection⁣^∗∗^

DRC	2001	> 31	7	1	—	Contact	Lymphadenopathy, conjunctivitis, pharyngitis and pulmonary failure, pustular lesions on the palms, soles, face, trunk and arms, coinfection⁣^∗^
Illinois, Indiana, Kansas, Missouri, Ohio and Wisconsin	2003	1–51	33	0	26 Vaccinated	Consumption of animal meat	Macular rash, cervical and tonsillar adenopathy, diffuse lesions in the oropharynx, general malaise, myalgia, respiratory symptoms and lymphadenopathy

DRC	2001–2004	> 35	51	—	—	—	Coinfection⁣^∗^

Wisconsin	2003	3–48	19	0	—	Occupational exposure with animals	Vesiculopustular rashes, headache, sweats, fever, chills, sore throat, cough and lymphadenopathy

Sudan	2005–2006	0–50	16	0	—	Contact	Vesiculopustular rash, lymphadenopathy, fever, cough, inflammation of nasal mucous membranes

North and South Kivu	2011–2014	1.5–28	3	0	Unvaccinated	Consumption of animal meat	Facial and oropharyngeal extended to arms, trunk and legs

DRC	2013	0–72	50	10	5	Familial and community contact	High fever, vesicular-pustular rash on the palms and soles, lymphadenopathy

DRC, South Sudan	2015-2016	1–9	14	3	—	Contact with animal meat	Fever and headaches, vesiculopapular with umbilications, pruritic, cervical adenitis, severe facial oedema, bilateral conjunctivitis, pulmonary oedema, malaise, myalgia, cervical and inguinal lymphadenopathy

South Sudan	2016	1–58	26	2	5 Vaccinated and 19 unvaccinated	Consumption of animal meat	Fever and vesiculopustular skin rashes, dysphagia, myalgia, headaches, pruritus and cervical and/or inguinal adenopathy

Nigeria	2017	6–46	42	1	—	Contact	Fever, generalized rash, headache, malaise, sore throat, papulopustular rashes on the trunk, face, palms and soles of the feet, oral and nasal mucosal lesions and ulcers and accompanying generalized lymphadenopathy, coinfection⁣^∗^, ⁣^∗∗^, ⁣^∗∗∗^

DRC	2017	—	7	6	—	Contact	Fever, vesiculopustular rash extending to hand palms and soles of the feet

UK	2018	—	5		—	Consumption of animal meat	Fever, lymphadenopathy and maculopapular rashes, scrotal lump

Nigeria	2017-2018	2 days–50 y	122	7	—	Contact	Coinfection⁣^∗∗^, secondary bacterial infection, fever, followed by a vesiculopustular rash occurring mostly on the face, palms and soles of feet

UK	2021	< 1 y	3		—	Contact and travel history	Coinfection⁣^∗∗∗∗^, afebrile and all skin lesions had crusted, vesicular rash

Brazil	2022	—	8164	10	—	Contact	Fever; rash; headache; adenomegaly; myalgia asthenia; backache; genital lesion; sore throat; localized lymphadenopathy; nausea; arthralgia; proctitis; penile oedema; cough; mucosal lesion; photosensitivity; haemorrhage; diarrhoea; conjunctivitis, coinfection⁣^∗∗∗^^,#,##,###^

Dallas, Texas	2021	—	35	—	—	Contact and travel history	Pustular and purulent rashes, fever, vomiting, diarrhoea

Portugal	2022	20–51	96	—	—	MSM, travelling, animal contact (two cases with cats and one case with pigs)	Genital white-coloured lesions, maculopapular or pustular lesions, anogenital complaints (including ulcers), asthenia, myalgia, backache, headache, inguinal lymphadenopathy, asthenia, anal vesicles, cervical lymphadenopathy, axillary lymphadenopathy, coinfection⁣^∗∗^

UK	2022	< 59	92	—	—	Sexual contact (gay or bisexual or MSM), other contact, travel history	—

Netherlands	2022	23–64	31	—	—	MSM	—

Singapore	2022	25–54	15	—	—	Travel history, contact	Anogenital and extragenital skin lesions, headache, anal discomfort and myalgia, lymphadenopathy, demonstrating vesicles, pustules, erosions and scabs, acute vesicular rash

Bahia, Brazil	2022	37 and 31	2	—	—	Oral sex, MSM	Lymphadenopathy, papular lesions on the skin, glans and scrotal sac, coinfection⁣^∗∗^

Spain	2022	20–60	1256	—	—	MSM, heterosexual, LGTBIQ	Fever, asthenia, muscle pain, throat pain, headache, anogenital rash, disseminated rash in locations other than anogenital or oro-/peribuccal, lymphadenopathy, oro-/peribuccal rash

Milan, Italy	2022	—	4	—	—	—	Vesicular lesions localized on the nose, ulceration, severe anal pain and peripheral leucocytosis, leucocytosis, thrombocytopenia, proctitis and severe perianal pain, coinfection⁣^∗∗^^,¶,¶¶,¶¶¶^

India	2022	—	3	—	—	Travel history	—

Italy	2022	34–43	33	—	3 Vaccinated	MSM	Coinfection⁣^∗∗^^,†,††^

Columbia	2022	—	2	—	Unvaccinated	Sexual contact, travel history	Itchy vesiculopustular rash, non-enhancing lesions of the bilateral basal ganglia, bilateral medial thalami, splenium and pons, central thoracic spinal cord and grey matter of the conus medullaris, encephalomyelitis, pelvic lymphadenopathy consistent with proctitis

Argentina	2022	—	3	—	—	—	Intergluteal and perianal lesions, genital ulcers, pustular lesions, maculopapular lesions, myalgia

India	2022	< 40	9	—	—	MSM	Vesiculopustular rashes in the anal and genital areas, systemic or local inflammation

India	2022	31 and 35	2	—	—	Travel history	Myalgia, pustular lesions, multiple cervical and inguinal lymph nodes, dysuria and genital swelling, chills, myalgia, backache and headache

Spain	2022	0.7–17	4663	2	—	Tattoo and piercing studio, sexual contact	Encephalitis, lymphadenopathy, asthenia, sore throat, myalgia, vomiting, diarrhoea

Florence, Italy	2022	45 and 59	2	—	—	MSM	Lesions in the oral mucosa, trunk, nipple, fever, malaise, sore throat and cervical lymphadenopathy, coinfection⁣^∗∗^^,^⁣^∗∗∗^^,##,††^

Hamburg	2022	20–67	179	—	32 Vaccinated and 119 unvaccinated	MSM, heterosexual	Coinfection⁣^∗∗^

Northeastern Nigeria	2022	< 40	21	—		Physical contact and infestations with rodents	Fever, rashes, malaise inguinal and cervical lymphadenopathy

USA	2022	32 and 37	2		Unvaccinated	MSM, sexual contact with multiple partners	Cervical and bilateral inguinal lymphadenopathy, chills, penile lesions and ulceration, chest pain and dysponea, myocarditis, coinfection⁣^∗∗∗^

New York, USA		22 and ?	2	—	—	Sexual contact	Myalgias, fatigue, fever, penile, perianal, vaginal and cervical lesions

Saudi Arabia	2022	24–41	7	—	—	Travel history	Penile lesions, finger paronychia, oral vesicles, pustule, umbilicated pustule and lymphadenopathy

Japan	2022	30	2	—	—	MSM (multiple partners), travel history	Cervical and inguinal lymphadenopathy, myalgia, stomatitis lesions, mucosa of the lower lips

Vietnam	2022	35 and 38	2	—	—	Travel history, sexual contact	Maculopapular lesion, fever, headache, chills, cough, sore throat, muscle pain and tiredness, coinfection⁣^∗^

Los Angeles, California, USA	2022	18–50	118	—	—	Sexual transmission, sharing clothing, food, utensils, dishes, bathrooms and razor blades; and shared shelter	Coinfection⁣^∗∗^, rashes, malaise, lymphadenopathy, headache, myalgia, chills, pruritus, vomiting or nausea, rectal pain, cough, abdominal pain, pus or blood on stools, rectal bleeding, conjunctivitis, tenesmus

Sudan	2022	—	120	18	Unvaccinated	Human-to-human transmission and direct contact in the refugee camps	

Chicago, Illinois	2023	23–49	48		40 Vaccinated	Sexual contact including MSM and bisexual	Genital, ocular and mucosal lesions, coinfection⁣^∗∗^

Northern California	2022	19 and 22	2	—	—	—	Severe diffuse pruritus

Taiwan	2023	—	79	—	—	—	—

Cologne	2022	12–80	368	—	168 Vaccinated	—	Skin and mucosal lesions, as well as general symptoms such as fever, night sweats, muscle/limb pain, lymphadenopathy and fatigue, coinfection⁣^∗∗^

Beijing, China	2023	24–45	37	—	—	Sexual contact (homosexual, MSM, bisexual)	Skin/mucosal lesions, coinfection⁣^∗∗^, lymphadenopathy, genital, perianal or perigenital skin rashes

Los Angeles, California, USA	2023	26–42	56	—	—	Sexual contact (homosexual, MSM, bisexual), travel history	Pruritus, fever, chills, coinfection^∗∗^, lymphadenopathy, rectal bleeding, genital lesions

Saudi Arabia	2023	20–39	381	—	—	Sexual contact	Coinfection⁣^∗∗^, fever, headache, cough, conjunctivitis, myalgia, Backpain, lymphadenopathy, exhaustion, rash (palms and soles, genitals, face, mouth, conjunctiva)

*Note:* MSM: Men having sex with men; coinfections reported with ⁣^∗^VZV, ⁣^∗∗^HIV, ⁣^∗∗∗^syphilis, ⁣^∗∗∗∗^COVID-19, ^#^chlamydia, ^##^gonorrhoea, ^###^genital herpes, ^¶^staphylococcal infection, ^¶¶^streptococcal infection, ^¶¶¶^bacterial cellulitis, ^†^hepatitis B and ^††^hepatitis C infection; ‘—' indicates that the data are not available or not known.

## Data Availability

All the generated data are presented in the manuscript.

## Nomenclature

APOBEC3: Apolipoprotein B mRNA–editing catalytic polypeptide-like 3
CDC: Centre for Disease Control and Prevention
CFR: Case-fatality ratio
COG: Conserved oligomeric Golgi
EEV: Extracellular enveloped virions
IEVs: Intracellular enveloped virions
MOPICE: Monkeypox inhibitor of complement enzymes
PHEIC: Public Health Emergency of International Concern
SPICE: Smallpox inhibitors of complement enzymes
VCP: Vaccinia complement control protein
